# The effect of spices *Coriandrum sativum* L., *Trigonella foenum-graecum* L., *Pimpinella anisum* L., and their combinations on growth performance, carcass trait, and hematobiochemical parameters in broiler chicken

**DOI:** 10.14202/vetworld.2022.1821-1826

**Published:** 2022-07-26

**Authors:** Samira Meradi, Ahmed Messaï, Miloud Aouachria

**Affiliations:** 1 Department of Agricultural Sciences, Promotion of Innovation in Agriculture in Arid Regions Research Laboratory, DEDSPAZA (Diversity of Ecosystèms and Systèmes Dynamiques of Agricoles Production en Zones Arides Laboratory), University Mohamed-Khider of Biskra, P.O. Box 145 RP, Biskra 07000, Algeria; 2 Scientific and Technical Research Centre on Arid Regions (CRSTRA), Campus Universitaire, Med Kheider, BP 1682 RP, Biskra 07000, Algeria

**Keywords:** broilers, blood parameters, natural feed additive, performances, spices seed

## Abstract

**Background and Aim:**

The incorporation of herbs and species has been shown to enrich the food with antioxidants and bioactive antimicrobial compounds, thereby preserving the safety and productivity of broiler chicken production. This study aimed to determine the effects of three phytogenic feed additives (PHT) on certain zootechnical and hematobiochemical parameters in broiler chickens. *Coriandrum sativum L*. (coriander), *Pimpinella anisum* L. (green anise), and *Trigonella foenum-graecum L*. (fenugreek) were used to formulate the PHT.

**Materials and Methods:**

A total of 360 1-day-old Cobb broilers for 42 days were randomly assigned to four dietary treatment groups: A control group (CTLG) and three groups fed a basal diet supplemented with 3% of coriander (PHT1G), 3% of a combination 50% coriander-50% fenugreek (PHT2G), and finally, 3% of a combination 50% coriander-50% green anise (PHT3G), respectively, and each experimental group included three repetitions of 30 birds. Zootechnical parameters, carcass productivity, and hematobiochemical properties were measured.

**Results:**

The birds in the PHT3G had the greatest body weight and organ weight (p < 0.05). However, the weight of abdominal fat remained unchanged. The same group of broilers had a significantly (p < 0.05) higher lymphocyte level of 120.10^3^/μL, followed by the PHT2G, which had 80.10^3^/μL. The levels of monocytes in the PHT2G and PHT3G were 66.10^3^/μL and 60.10^3^/μL, respectively. Regarding granulocytes, we observed 200.10^3^/μL in the PHT2 group and 102.10^3^/μL in the PHT3G. There was a statistically significant difference (p < 0.05) between the uric acid levels of the PHT1G, PHT2G, and PHT3G, with 50.4 mg/L, 59.84 mg/L, and 47.29 mg/L, respectively. All experimental groups had significantly lower uric acid concentrations than the control group (84.36 mg/L).

**Conclusion:**

The use of phytogenic feed additives may positively affect both weight gain and hematobiochemical parameters in broiler chicken, particularly the levels of various white blood cell subtypes and the uric acid rate.

## Introduction

Herbs and spices have been extensively studied as an alternative to antibiotics and as growth promoters in poultry diets [1]. It is advantageous to feed medicinal herbs to poultry in response to consumer demands and legislative restrictions on the use of antibiotic growth promoters and ionophores in modern intensive poultry production [2]. Consumers are interested in meat quality that is produced by sustainable livestock products that are free of chemicals that are harmful to health and have superior sensorial and preservation properties [3].

The effects of phytogenic are linked to their phytochemical constituents. The bioactive molecules increase chicken production potential by enhancing poultry immunity [4] and enhancing the digestive system. They also maintain a healthy gut microflora and intestinal uptake [5]. These benefits can be realized by incorporating medicinal plants into the feed or drinking water of broiler breeding [6]. The incorporation of additives also enriches food with antioxidants, bioactive antimicrobial compounds, and numerous spices. *Coriandrum* is a medicinal and spice plant whose leaves, seeds, and fruits possess numerous beneficial biological properties, including antimicrobial, antioxidant, and anti-inflammatory activities [7].

Considerable research indicates that the inclusion of coriander seed in poultry feed improves zootechnical performance, carcass yield, blood biochemical profile, and mineral content of chicken meat [7]. Coriander seed powder has been utilized as an alternative to antibiotics against Newcastle and infectious bronchitis in chicken feed, according to Hosseinzadeh *et al*. [8]. However, fenugreek is abundant in bioactive compounds such as flavonoids, phenols, saponins, alkaloids, and other bioactive compounds [9]. It has many intriguing bioactive properties, including antimicrobial, antioxidative, antifungal, and antiviral properties, as well as digestive stimulation and immunomodulation [10]. Furthermore, recent studies on broiler chickens indicate that fenugreek supplementation significantly reduces blood cholesterol and glycemic levels, stimulates the immune system, and improves plasma total protein and globulin [11]. Furthermore, green anise has antimicrobial properties. Its active components improve the biochemical profile of blood, increase calcium and phosphorus serum levels, and enhance the albumin/globulin ratio, thereby enhancing chickens’ resistance to various stress factors [12]. Green anise increases immune response, improves meat quality, reduces gut bacteria, and improves morphological characteristics, according to Gupta *et al*. [13]. Individually or in combination, phytogenic herbs and species preserve the safety and production of broilers [14]. Although numerous studies have been conducted on the use of phytogenics as growth promoters in animal production, it is still worthwhile to investigate the benefits of these natural substances.

This study aimed to assess the effects of local spices, including *Coriandrum sativum* L. (coriander) and its combination with *Pimpinella anisum* L. (green anise) and *Trigonella foenum-graecum* L. (fenugreek) on growth performance, carcass traits, and hematobiochemical parameters in broiler chickens.

## Materials and Methods

### Ethical approval

All procedures performed in this research were approved by the Scientific and Technical Research Centre on Arid regions (CRSTRA)-University of Briskra (Approval no. 04042021CRSTRA).

### Study period and location

The study was conducted during March and April 2021 at a broiler farm of Department of Agricultural Sciences, University Mohamed-Khider of Biskra, Algeria.

### Animals and their diets

A total of 360 1-day-old Cobb 500 broilers were purchased from a commercial hatchery and raised in litter floor pens at the Department of Agricultural Sciences, University of Biskra, Algeria. The initial body weight (BW) of the chicks was 47.33 ± 0.10 g.

Four dietary treatment groups were formed: A control group (CTLG) fed a basal diet and three groups fed a basal diet supplemented with feed additives for 42 days. A group supplemented with 3% coriander (PHT1G), a group supplemented with 3% of a mixture of 50% coriander and 50% fenugreek (PHT2G), and a group supplemented with 3% of a mixture of 50% coriander and 50% anise (PHT3G). Each experimental group consists of 30 birds repeated 3 times.

Feed and water were *ad libitum*. According to NRC [15] recommendations, isocaloric and isonitrogenous rations were formulated. The ingredients and nutrient compositions of the basal diet are summarized in Table-1.

**Table 1:** Ingredients and nutrient compositions of basal diet.

Basal diet	Starter (0–15 days)	Grower (16–42 days)
Ingredients		
Maize	63	65
Soybean meal	29	27
Wheat bran	5	5
Phosphate	1	1
Calcaire	1	1
CMV	1	1
Chemical composition		
EM (Kcal/kg)	2820.57	2844.97
DM %	92.73	92.65
Crude proteins (%DM)	22.06	20.75
Ether extract (%DM)	2.06	2.08
Ash (%DM)	5.02	4.52
Fiber (%DM)	4.33	4.02
Calcium (%DM)	6.97	1.31
P disponible	0.69	0.63
Lysine (%CP)	3.23	3.55
Méthionine (%CP)	1.34	1.44

DM=Dry matter, CP=Crude protein, CMV=complex mineral vitamin, EM=Metabolizable energy

### Plant material

We used Biskra local seeds of coriander, green anise, and fenugreek; these Bioressources were identified by a team of researchers in the Scientific and Technical Research Centre on Arid Regions. The samples were deposited at the CRSTRA under the numbers: (003CRSTRA0003) for coriander, (011CRSTRA0006) for fenugreek and (003CRSTRA0011) for green anise. Within 6 days after collection, seeds were cleaned, air-dried, and stored under correct conditions until used as phytogenics. The harvests of the three types of seeds were carried out in 2020 in Biskra Province of Algeria.

### Growth performances and carcass traits

The zootechnical parameters such as Live body weight [LBW], average daily gain [ADG], and feed conversion ratio [FCR]) were measured. The feed intake and conversion ratio were determined daily by weighing diets distributed and feed refused. However, weekly bird weights were taken to determine the daily feed intake and the FCR. However, weekly birds were weighed to calculate the average daily weight gain. At the end of the experiment period (day 42), 10 subjects per replicate from each group were selected at random and individually weighed to determine their LBW. The chosen birds were sacrificed and eviscerated. The carcasses and internal organs were weighed, including the liver, proventriculus, gizzard, and small intestine. We measured the abdominal fat, the breast, and the leg (thigh + drumstick). The carcass yield was expressed as a proportion of the total body mass.

### Hematobiochemical parameters

On day 39, 10 subjects were selected at random from each group. Blood samples were obtained from the wing vein. The biochemistry of the blood (levels of glycemia, total cholesterol, total proteins, globulin, and albumin) and, the cellular composition of the blood (red blood cells, lymphocytes, monocytes, and granulocytes) were analyzed using an automated blood cell counter (ERMA PCE 210, Japan).

### Statistical analysis

Statistical Package for the Social Sciences software version 25.0 (IBM Corp., NY, USA) was used to analyze the data using analysis of variance, followed by a comparison of means based on the Newman and Keuls tests. The difference was considered significant when p < 0.05.

## Results

### Growth performance

The analysis of the results shows that during a period of 42 days, the highest LBW and the ADG were recorded in PHT3G with 2967 g and 70.64 g, respectively. However, feed intake did not differ (affect [p < 0.05]) between PHTG3, PHTG2, and CTLG, which are 4207 g, 4187.06 g, and 4083 g, respectively. The best FCR was not significantly different (p < 0.05) between the CTLG, PHTG2, and PHTG3: 1.40, 1.46, and 1.47, respectively (Table-2).

**Table 2:** Growth performances in experimental groups.

Parameters	CTLG	PHT1G	PHT2G	PHT3G	ESM
LBW (g/bird)	2945.02^b^ ± 13.2	2919.58^a^ ± 92.88	2927.62^a^ ± 148.89	2966.98^c^ ± 37.38	51.93
ADG (g)	70.12^a^ ± 0.31	69.51^a^ ± 2.21	69.71^a^ ± 3.54	70.64^a^ ± 0.89	2.31
Feed intake (g/bird)	4083.44^a^ ± 4.32	4859.95^b^ ± 92.69	4187.06^a^ ± 125.47	4206.65^a^ ± 93.84	1.252
FCR	1.38^a^ ± 0.007	1.67^b^ ± 0.04	1.43^a^ ± 0.03	1.42^a^ ± 0.04	0.019

LBW=Live body weight, ADG=Average daily gain, FCR=Feed conversion ratio.^a,b,c,d^ Means in the same row with different letters show significant differences (p < 0.05) among dietary

### Carcass traits

The productivity of carcasses is summarized in Table-3. The statistical analysis revealed that the birds were fed PHTG3 and those in the CTLG had greater LBW and eviscerated carcasses. However, PHTG2 produced the highest carcass yield at 75.44%, followed by PHTG3 at 73.16%. Indeed, PHTG1 and PHTG2 improved the percentage of breast composition by 31.70% and 31.22%, respectively. With PHTG3, the percentage of the leg has improved by 9.73% more than with the other experimental diet. The phytobiotic compounds PHTG2 and PHTG3 did not influence the abdominal fat. In contrast, we noted that the weight of various visceral organs increased significantly (p < 0.05) with PHTG3.

**Table 3:** Carcass components.

Parameters	CTLG	PHT1G	PHT2G	PHT3G	ESM
LBW at slaughter (g)	3325^b^ ± 240.42	3217.50^b^ ± 357.09	2810^a^ ± 28.28	3290^b^ ± 275.77	
Eviscerated carcass (g)	2405^b^ ± 197.99	2315^b^ ± 0.296.98	2120^a^ ± 70.71	2407.5^b^ ± 208.60	0.198
Carcass yield (%)	72.33	71.96	75.44	73.16	0.198
Breast (g)	987^b^ ± 102.53	1020^c^ ± 28.28	877.5^a^ ± 17.68	930^b^ ± 183.85	
Breast (%)	29.68	31.70	31.22	28.27	0.300
Leg (g)	310^b^ ± 21.21	305^b^ ± 14.14	255^a^ ± 14.14	320^b^ ± 49.50	
Leg (%)	9.32	9.48	9.07	9.73	0.525
Abdominal fat (g)	27^a^ ± 4.24	51^b^ ± 1.41	33^a^ ± 7.07	40^a,^ ^b^ ± 8.49	
Abdominal fat (%)	0.81	1.59	1.17	1.21	0.112
Heart (g)	14^a^ ± 1.41	14.06^a^ ± 1.41	15^a^ ± 1.44	17^a^ ± 2.83	
Heart (%)	0.42	0.44	0.53	0.51	0.300
Liver (g)	59^a^ ± 7.07	78^b^ ± 26.87	52^a^ ± 7.07	66.5^a,b^ ± 9.19	
Liver (%)	1.77	2.42	1.85	2.02	0.252
Proventriculus (g)	9^b^ ± 0.01	10^b^ ± 2.83	7.5^a^ ± 0.71	9.05^b^ ± 0.03	
Proventriculus (%)	0.27	0.31	0.27	0.27	0.537
Gizzard (g)	47.5^a^ ± 20.51	56^b^ ± 9.90	46^a^ ± 5.66	50^a,^ ^b^ ± 2.83	
Gizzard (%)	1.43	1.74	1.64	1.51	0.139

^a,b,c,d^ Means in the same row with different letters show significant differences (p < 0.05) among dietary

### Hematobiochemical parameters

The glycemic levels of all groups were unaffected by phytobiotic compounds (p < 0.05). However, the total cholesterol levels in the PHTG1, PHTG2, and PHTG3 were significantly lower than in the CTLG (p < 0.05): 0.92 g/L, 0.95 g/L, and 1.05 g/L for PHTG3, PHTG1, and PHTG2, respectively. Total protein levels with PHTG1 and PHTG2 were higher than those of the control and PHTG3 (p < 0.005). Concerning globulin, the highest value was found in PHTG1 and PHTG2, but there was no significant difference (p < 0.05). The highest albumin concentration was found in PHTG2 followed by PHTG1 (12.19 g/L, 11.41 g/L, and 10.81 g/L). In terms of the ratio (albumin/globulin), the most significant value was found in PHTG3 with 0.86 and in CTLG with 0.76. The lowest ratios were observed in PHTG1 and PHTG2 in that order (0.37 and 0.43). Notably, the CTLG had the lowest red blood cell count, 3.48.10^6^/mm^3^, and the PHTG3 had the highest lymphocyte count, 120.10^3^/μL, followed by PHTG2 with 80.10^3^/μL. In both the PHTG2 and PHTG3, significant monocyte levels were observed at 66.10^3^/μL and 60.10^3^/μL, respectively. We observed 200.10^3^/μL for PHTG2 and 102.10^3^/μL for PHTG3 in granulocytes. The results of the hematobiochemical analysis are summarized in Table-4.

**Table 4:** Hematobiochemical parameters.

Parameters	CTLG	PHT1G	PHT2G	PHT3G	ESM
Blood biochemistry					
Glycemia (g/L)	2.21^a^	2.09^a^	2.58^a^	2.37^a^	0.622
Total cholesterol (g/L)	1.19^a^	0.95^a^	1.05^a^	0.92^a^	0.431
Total protein (g/L)	26.25^a^	39.37^b^	40.48^b^	20.5^a^	0.655
Uric acid (uricemia) (mg/L)	84.36^c^	50.4^a^	59.84^b^	47.29^a^	0.623
Creatinine level (mg/L)	2.3^a^	2.7^a^	2.2^a^	2.2^a^	0.222
Globulin (g/L)	14.84^a^	28.56^b^	28.29^b^	10.97^a^	0.516
Albumin level (g/L)	11.41^a^	10.81^a^	12.19^b^	9.53^a^	0.122
Albumin/globulin	0.76^b^	0.37^a^	0.43^a^	0.86^b^	
Hematology					
Red blood cell/mm^3^	3.48.106^a^	4.08.106^a^	3.95.106^a^	3.82.106^a^	0.687
Lymphocytes/μL	62.103^a^	60.103^a^	80.103^b^	120.103^c^	0.852
Monocytes/μL	12.103^a^	18.103^a^	66.103^b^	60.103^b^	0.866
Granulocytes/μL	66.103^a^	72.103^a^	200.103^c^	102.103^b^	0.874

^a,b,c,d^ Means in the same row with different letters show significant differences (p < 0.05) among dietary

## Discussion

### Growth performances

Our findings indicate that the highest zootechnical parameters were achieved in diets supplemented with the phytogenic feed additive PHT3. We observed an increase in LBW and ADG.

Our growth performance results are consistent with results obtained by Samani *et al*. [16]. By supplementing broiler chicken with coriander seeds, fenugreek, and green anise, the authors confirmed an increase in BW, feed intake, and a decrease in the FCR. Moreover, the biological effects are dependent on the doses and ratios of the various natural products used [17, 18]. Phytogenic feed additives augment the palatability of feed. Herbs, spices, and their active compounds positively affect the oronasal system when used as food condiments. Oronasal sensing prompts the digestive tract to accept food and stimulates digestive secretions [19]. According to Lillehoj *et al*. [20], there is a significant relationship between specific bacterial populations and certain phytochemicals in the gut of domesticated animals, which promotes increased productivity. Natural compounds are capable of modulating the microbiome of the gastrointestinal tract and enhancing animal welfare and production.

In contrast, Lee *et al*. [18] reported that the effects of phytogenic seed as a feed additive powder at different levels on bird performance were insignificant. This effect was explained by Khubeiz and Shirif [7] as a result of the high nutritional quality of the basal diet and environmental conditions; diets containing highly digestible ingredients inhibit the proliferation of bacteria in the gastrointestinal tract because there is no substrate to support bacterial growth. In our study, the enhancement of growth performance by the coriander and green anise-based phytogenic feed additive [19] may have resulted from the linalool-mediated enhancement of palatability and digestive enzymes.

### Carcass trait

According to our findings, PHTG3 improved both LBW and eviscerated carcass. However, PHTG2 produced the highest carcass yield at 75.44%, while PHTG3 improved the proportion of legs to 9.73%. The phytobiotic compounds did not affect the abdominal fat. While statistical results demonstrated that the weight of various visceral organs increased significantly (p < 0.05) with PHTG3, in fact, the effect of phytobiotics on carcass and internal organ performance is consistent with the findings of Giannenas *et al*. [21], who found that natural products increase the feed intake and live weight of birds, which affect carcass yield, liver, heart, and gastrointestinal function. Al-Homidan *et al*. [11] reported that the inclusion of fenugreek in chicken diets has no negative effect on chicken performance, carcass weight, or internal organ weight. Bioactive compounds from herbs and spices have beneficial effects on animal digestion, which can influence weight gain because it involves metabolic and immune regulation [22]. A great deal of research has centered on meat quality and the oxidation process. The oxidation of lipids and proteins is recognized as a major threat to the quality of poultry products. The incorporation of phytochemicals into animal feed or directly into meat products is a viable solution [23]. In our study, the significant improvement of carcass parameters could be explained by the stimulatory effects of digestive enzyme secretions, which induce better absorption of nutrients from the digestive tract, such as amino acids [24]. In addition, the antioxidants and phenolic compounds in vegetable products enhanced the breast meat of broilers by 1.2% [25].

### Hematobiochemical parameters

In this study, dietary supplementation with phytogenic feed additives decreased total cholesterol and increased total protein levels in the blood of broilers in a linear fashion. At PHTG2 and PHTG3, the level of monocytes and red blood cells increased significantly. Our hematobiochemical parameter results are consistent with those obtained by Saeid and Al-Nasry [26], who observed that the glycemia level varied between 1.09 g/L and 2.13 g/L with varying levels of coriander seeds in broiler feed. Adil *et al*. [27] reported that incorporating fenugreek seeds into the diet of broilers decreased their blood cholesterol and glycemia levels. The presence of saponins and resins in fenugreek inhibits the absorption of bile acids and cholesterol in the gut, resulting in a decrease in blood cholesterol levels [28]. Brenes and Roura [19] have reported that the incorporation of phytogenic compounds into poultry feed improves pancreatic digestive enzymes.

According to Oueslati and Ghédira [29], numerous pharmacological and clinical studies have shown that fenugreek has antioxidant, cholesterol-lowering, and hypoglycemic effects due to the constituents of the seed, which include steroids (diosgenin), alkaloids (trigonelline), flavonoids (luteolin), coumarins, amino acids (hydroxy isoleucine), mucilages (galactomannan), volatile constituents, and fixed oil, among other substances. These molecules are measured to evaluate physiologic functions of the immune system and to track metabolic and nutritional diseases [18]. The incorporation of fenugreek and coriander decreased the level of uricemia, as observed by Saeid and Al-Nasry [26], whose findings are consistent with the results of this study. Al-Shammari *et al*. [30] demonstrated that the addition of anise seed powder to the drinking water of Hubbard Classic broilers significantly improves the blood profile; anise seed is a potent physiological promoter in broilers. Adil *et al*. [27] noted a significant increase in red blood cell volume and hemoglobin concentration (p < 0.05) when 10 g/kg of fenugreek was added to the diet of broilers. However, others noted that unlike the addition of fenugreek, laying hens’ serum metabolites and immune responses were not affected [16]. Saeid and Al-Nasry [26] confirmed that coriander seeds enhance the hematological composition of red blood cells, hemoglobin, and platelets in broiler chickens but found no difference in the white blood cells.

## Conclusion

This study demonstrated that the phytobiotic compounds PHTG2 and PHTG3 have greater significance than coriander alone. However, PHTG3 was the most intriguing compound, followed by PHTG2. The LBW, ADG, feed intake, FCR, and carcass yield have all increased. The proportion of the breast and leg was increased. In fact, the phytobiotic compounds had no effect on the abdominal fat. The combination of *C. sativum* L., *P. anisum* L., and *T. foenum-graecum* L. has a positive effect on hematobiochemical parameters in broiler chicken, particularly increasing the number of various white blood cell types (lymphocytes, monocytes, and granulocytes). As phytogenic feed additives possess medicinal properties such as antimicrobial and antioxidant, they can promote safe poultry husbandry and could be recommended as supplements, according to the research.

## Authors’ Contributions

All authors contributed to the study conception and design. SM and AM: Experimentation, data collection, and data analysis. SM: Drafted and revised the manuscript. MA: Reviewed and edited the manuscript. All authors have read and approved the final manuscript.
